# Cost-effectiveness analysis of antipsychotics in reducing schizophrenia relapses

**DOI:** 10.1186/2191-1991-2-8

**Published:** 2012-04-10

**Authors:** Antonio J García-Ruiz, Lucía Pérez-Costillas, Ana C Montesinos, Javier Alcalde, Itziar Oyagüez, Miguel A Casado

**Affiliations:** 1 Catedra de Economia de la Salud y Uso Racional del Medicamento, Pharmacology and Clinic Therapeutic Department, University of Malaga, Malaga, Spain; 2 Carlos Haya General Hospital, Malaga, Spain; 3 Social Psychology Department, University of Malaga, Malaga, Spain; 4 Pharmacoeconomics and Outcomes Research Iberia, De la Golondrina 40A, Madrid 28023, Spain

**Keywords:** Schizophrenia, Relapse, Antipsychotic, Cost-effectiveness, Cost-utility, Spain

## Abstract

**Background:**

Schizophrenia is a severe form of mental illness which is associated with significant and long-lasting health, social and financial burdens.

The aim of this project is to assess the efficiency of the antipsychotics used in Spain in reducing schizophrenia relapses under the Spanish Health System perspective.

**Material and methods:**

A decision-analytic model was developed to explore the relative cost-effectiveness of five antipsychotic medications, amisulpride, aripiprazole, olanzapine, paliperidone Extended-Release (ER) and risperidone, compared to haloperidol, over a 1-year treatment period among people living in Spain with schizophrenia. The transition probabilities for assessed therapies were obtained from the systemic review and meta-analysis performed by National Institute for Health and Clinical Excellence (NICE).

**Results:**

Paliperidone ER was the option that yielded more quality-adjusted life years (QALYs) gained per patient (0.7573). In addition, paliperidone ER was the least costly strategy (€3,062), followed by risperidone (€3,194), haloperidol (€3,322), olanzapine (€3,893), amisulpride (€4,247) and aripiprazole (€4,712).

In the incremental cost-effectiveness (ICE) analysis of the assessed antipsychotics compared to haloperidol, paliperidone ER and risperidone were dominant options. ICE ratios for other medications were €23,621/QALY gained, €91,584/QALY gained and €94,558/QALY gained for olanzapine, amisulpride and aripiprazole, respectively. Deterministic sensitivity analysis showed that risperidone is always dominant when compared to haloperidol. Paliperidone ER is also dominant apart from the exception of the scenario with a 20% decrease in the probability of relapses.

**Conclusions:**

Our findings may be of interest to clinicians and others interested in outcomes and cost of mental health services among patients with schizophrenia.

Paliperidone ER and risperidone were shown to be dominant therapies compared to haloperidol in Spain. It is worthwhile to highlight that schizophrenia is a highly incapacitating disease and choosing the most appropriate drug and formulation for a particular patient is crucial.

The availability of more accurate local epidemiological data on schizophrenia would allow a better adaptation of the model avoiding some of the assumptions taken in our work. Future research could be focused on this.

## Background

Schizophrenia is a severe form of mental illness that has varying presentations. As a chronic disease, it is associated with significant and long-lasting health, social and financial burdens, not only for patients but for their families, other caregivers [1] and wider society [2,3].

According to WHO estimates, schizophrenia affects approximately 24 million people worldwide [4]. The most recent publications estimate that the median incidence of schizophrenia varies from 15.2 [5] to 20.0 per 100,000 population/year [6], although it is higher in the 15-35 year-old age group (7 per 1,000 population) [7,8]. There are no recent epidemiological data concerning schizophrenia in Spain. Spain does not have a national registry that would make it possible to know the exact number of individuals with schizophrenia, although regional studies estimate the prevalence at 0.6-0.8% for the adult population (17 years of age and older) [9]. Available estimates from 1995 show that incidence of schizophrenia was 1.9 per 10,000 inhabitants per year for people between the ages of 15 and 54 years [10].

Conventional antipsychotic medications (chlorpromazine and haloperidol) emerged 50 years ago as the first tools on the management of schizophrenia, in concert with other important interventions, such as psychosocial and economic support and rehabilitation efforts. However, the unrivalled role of conventional antipsychotic medications has been continuously challenged by the wide range of adverse effects of these medications. Over the last 15 years, several new atypical antipsychotic medications have been introduced, including sertindole, risperidone, olanzapine, quetiapine, amisulpride, ziprasidone, aripiprazole and paliperidone [11]. In general, the new antipsychotics have shown themselves to be at least comparable in efficacy to conventional antipsychotics but with superior tolerability and a more favourable adverse effect profile, providing less extrapyramidal side effects than conventional treatment [8].

However, despite the availability of new antipsychotics, 20-30% of patients have an inadequate response to medication with 15-20% relapsing each year [2,12]. Compliance is one of the factors associated with relapse [13]; however, some patients relapse while taking maintenance medication [14].

Relapse has wider implications for the family in general, for the provision of medical and social facilities and from a health economic perspective [15]. In terms of quality of life, it has been shown to be associated with lower quality of life than in other stable medical conditions [16]. Onset of the disease in late adolescence or early adulthood [2] together with difficulties in employment and the social stigma associated with schizophrenia could be considered the main drivers of the changes in quality of life seen in these patients [16,17].

Additionally, schizophrenia has been shown to place a substantial economic burden on both the health care system and society worldwide due to its potentially large demands on the healthcare system [18]. The full cost of schizophrenia is high, although this is rarely appreciated by health care decision makers or other stakeholders [3].

In Spain, drug's reimbursement is a central national decision. However, there is a strong territorial decentralization of health jurisdictions in the Autonomous Communities. Therefore, the final drug financing is an Autonomous Regions' responsibility covering almost 100% of population [19]. That means that caring of schizophrenia patients is covered by the public system. In the practice, schizophrenia is diagnosed and followed-up by a psychiatrist at a hospital level, after the patient is being referred by a general practitioner. First drug prescription is usually made by the specialist, but it is required that the patient attends to a primary care level asking for drug prescription in a funded way. In the global economic crisis environment we face, the efficient use of available healthcare resources is required to maximise health benefits for people with schizophrenia and, at the same time, reduce the emotional distress and financial implications of the condition to society [18]. Use of atypical antipsychotics could increase the total cost of the disease as a result of their relatively high price. However, their effectiveness results in reduced hospital stays, thus potentially decreasing total outlay. Indeed, these more expensive treatments could be cheaper for society in the long run [3]. The efficiency, understood as combined measure of efficacy and cost, of treatments for schizophrenia is one key parameter in the decision-making process [20].

Published economic evaluations suggest that atypical (second-generation) antipsychotics are cost-effective when compared with conventional (first-generation) therapy [21]. The aim of this project was to assess the efficiency of the antipsychotics used in Spain to reduce schizophrenia relapses under the National Health System perspective.

## Methods

### Model design

**Figure 1:**
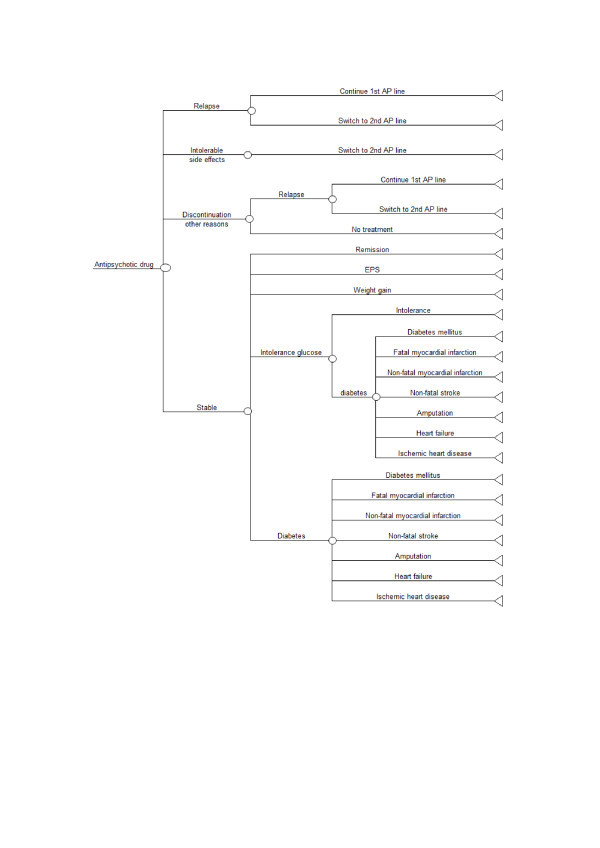
**Schematic decision tree model for antipsychotic drugs in patients with schizophrenia**.

A decision-analytic model was developed using TreeAge software (^© ^2009 TreeAge Software, Inc. - Decision Analysis Software) to explore the relative cost-effectiveness of antipsychotic medications for people with schizophrenia in Spain. Figure 1 shows a schematic representation of the decision tree used.

The model compares oral amisulpride, aripiprazole, olanzapine, paliperidone Extended-Release (ER) and generic risperidone with generic oral haloperidol (primary comparator) in the treatment of patients with schizophrenia. Zotepine was discarded for not being marketed in our country.

According to the model structure, six hypothetical cohorts of people with schizophrenia in remission were initiated on each of the six oral antipsychotic medications assessed (first-line antipsychotic).

Patient could stop the first line antipsychotic when relapsing or due to the development of intolerable side effects and switch to a second line antipsychotic. People who stopped the first line antipsychotic for any other reason were assumed to do so abruptly and were moved to the no treatment group. These people remained without antipsychotic treatment until they experienced a relapse. People discontinuing treatment due to side effects or for other reasons were assumed not to experience relapse in the remaining time after the first line antipsychotic discontinuation occurred. All patients experiencing a relapse stopped any antipsychotic they had been receiving while in remission and were treated for the acute episode; after achieving remission, they either returned to their previous antipsychotic medication with the goal of promoting recovery (50% of them) or switched to a second line antipsychotic drug (the remaining 50%).

The first line antipsychotic described in the model structure was one of the six (amisulpride, aripiprazole, olanzapine, paliperidone ER or risperidone) oral antipsychotics evaluated in the analysis. The second line antipsychotic following the first line was haloperidol; the second line antipsychotic following haloperidol was olanzapine.

The model assessed the relative cost-effectiveness between the first line antipsychotics only. The purpose of incorporating medication switching in the model structure was to check the impact of the lack of effectiveness in relapse prevention (expressed by relapse rates), intolerance (expressed by discontinuation rates due to side effects) and unacceptability (expressed by discontinuation rates due to other reasons) of the first line antipsychotics on cost and health outcomes and to present a more realistic sequence of events related to treatment of people with schizophrenia with antipsychotic medication.

The model assumes that four types of side effects could be experienced by patients: extra-pyramidal symptoms (EPS) [22], clinically significant weight gain (increase in weight of at least 7% from baseline) and glucose intolerance or diabetes as a representative feature of the metabolic syndrome. It must be noted that acute EPS did not include cases of tardive dyskinesia [9,23].

### Model probabilities

Probabilities for each one of the assessed therapies were obtained from the systemic review and meta-analysis performed by NICE [18]. This systematic review was developed based on the results of 17 randomized clinical trials (RCT) including 3,535 subjects. All the analyzed RCTs provided information concerning the three main parameters considered in the economic model: relapse, treatment discontinuation due to side effects and treatment discontinuation for other reasons.

Table 1 details the probabilities used in the model. The probabilities that treatments are best in reducing relapse over 52 weeks were re-calculated from the probabilities used in the NICE model, with the exception of zotepine.

**Table 1:** Mean values of probabilities employed in the decision tree

Strategy	Probabilities of relapse over 52 weeks	Probabilities of discontinuation of treatment over 52 weeks		Probabilities that treatment is best in reducing relapse over 52 weeks (reassessed excluding zotepine)	Probabilities of adverse events				Probabilities of Diabetes Mellitus complications					
				
		Intolerable side effects	Other reasons		EPS	Weight gain	Glucose intolerance	Diabetes Mellitus	Amputation	Fatal myocardial infarction	Non-fatal myocardial infarction	Non-fatal stroke	Heart failure	Ischaemic heart disease
Amisulpride	0.2988	0.0554	0.2435	0.084	0.3163	0.3175	0.2381	0.0317	0.0023	0.0042	0.0130	0.0039	0.0040	0.0157
						
Aripiprazole	0.2742	0.1582	0.3520	0.119	0.2258	0.1516	0.1167	0.0156						
						
Haloperidol	0.3317	0.0922	0.2516	0.035	0.5367	0.2000	0.1500	0.0200						
						
Olanzapine	0.1996	0.0783	0.2730	0.152	0.2336	0.4172	0.3129	0.0417						
						
Paliperidone ER	0.1625	0.3287	0.3848	0.525	0.2569	0.2123	0.1592	0.0212						

Risperidone	0.2761	0.0994	0.1761	0.086	0.3546	0.2141	0.1606	0.0214						

EPS: Extra-Pyramidal Symptoms)

ER: Extended-Release

Annual probability of transition of impaired glucose intolerance to diabetes = 0.0196

### Time horizon, perspective and discount rate

The model was run from a third party payer perspective (National Health System) during a one-year timeframe. This period was chosen because clinical data on relapse on discontinuation were taken from trial lasting between 26 and 104 weeks. No robust evidence exists to confirm that extrapolation of effectiveness data reflects the long-term effectiveness of antipsychotic medication and its impact on the course of schizophrenia in real life.

The time horizon is less than one year thus no annual discount rate was applied [24].

### Cost-effectiveness analysis

For each treatment, the average benefit in terms of Quality adjusted life year (QALY) gained and the average cost was calculated. The average cost-effectiveness ratio of each treatment was obtained.

Efficiency was established from the Incremental Cost-Effectiveness Ratio (ICER), defined as extra cost per QALY gained [25] with any of the antipsychotics versus haloperidol:

$$\text{ICER}=\frac{\left(\text{Cost}\;\text{strategy}1)-(\text{Cost}\;\text{haloperidol}\right)}{\left(\text{Effectiveness}\;\text{strategy}1)-(\text{Effectiveness}\;\text{haloperidol}\right)}$$

A strategy is considered as cost-effective versus haloperidol when ICERs are below the cost-utility threshold acceptable in Spain (€30,000/QALY gained) [26,27]

A strategy is dominant in comparison to haloperidol, when effectiveness (in QALYs) is higher, and cost is lower than the compared drug. In the same way, a strategy is dominated when effectiveness is lower, and cost is higher.

### Utilities

To express outcomes in QALYs, the health states of the economic model needed to be linked to appropriate utility scores. Utility scores represent the HRQoL associated with specific health states on a scale from 0 (death) to 1 (perfect health); they are estimated using preference-based measures that capture people's preferences on, and perceptions of, HRQoL in the health states under consideration [18].

Utility scores for remission and relapse were derived from those published by Lenert et al [28]. Utilities for acute EPS and weight gain were calculated by multiplying the remission utility value by the expected decrement in utility reported by NICE (0.888 for acute EPS and 0.959 for weight gain) [18], which was estimated from the number of people endorsing the presence of each side effect, as reported in a paper by Lenert and colleagues [28].

Utilities owing to diabetes mellitus were taken from a national study carried out in Spain based on EQ-5D tariffs [29]. Utilities arising from complications from diabetes were also extracted from the literature [30]. Utility scores used in the model are detailed in Table 2.

**Table 2:** Utility scores

Status	Utility value	Comments
Remission	0.799 [28]	

Relapse	0.670 [28]	

Acute EPS (extra-pyramidal symptoms)	0.7095	Calculated by multiplying remission utility by expected decrement in utilities estimated in the NICE guidance [18] (0.888 for acute EPS and 0.959 for weight gain)
	
Weight gain	0.7662	

Diabetes Mellitus (controlled)	0.760 [29]	

Diabetes Mellitus complications	Disutility value [30]	

Amputation	-0.109	

Fatal myocardial infarction	1.000	

Heart failure	-0,108	

Ischaemic heart disease	-0.132	

Non-fatal myocardial infarction	-0.129	

Non-fatal Stroke	-0.181	

### Cost estimation

Costs considered in the model consisted of drug acquisition costs, inpatient stays due to relapse, outpatient primary and community care costs of treating adverse events and metabolic complications of antipsychotic treatment.

Patients under no treatment (following treatment discontinuation for reasons other than relapse or presence of intolerable side effects) were assumed to incur no costs until they experienced a relapse. Costs associated with baseline measurements and laboratory tests for monitoring purposes were omitted from the analysis, as they were estimated to be the same for all antipsychotic medications evaluated.

Drug acquisition costs were based on mean doses used as described by IMS (ICD codes F20) in Spain for each treatment (Table 3). Prices expressed in public prices (VAT included) per mg (price weighted by relative sales by put up) were obtained from the Pharmacist Official Council Catalogue [31].

**Table 3:** Acquisition costs of antipsychotic medications (€, 2009)

Strategy	Mean Daily Dose used (mg)	Price/mg (included VAT in €)	Daily Treatment Cost (€)	Annual cost (€)
Amisulpride	611.12	0.0086	5.2556	1,918.31

Aripiprazole	15.42	0.4215	6.4995	2,372.33

Haloperidol	8.70	0.0356	0.3097	113.05

Olanzapine	14.65	0.4382	6.4196	2,343.16

Paliperidone ER	6.40	0.8906	5.6998	2,080.44

Risperidone	5.39	0.5566	3.0001	1,095.03

ER: Extended-Release

The average cost of hospitalisation, including inpatient stay and pharmacological treatment for people experiencing acute episodes, was estimated from hospitalisation costs for people with schizophrenia, schizotypal and delusional disorders in Spain, from the disease-related group 430 (DRG-430, psychosis) [32]

Acute EPS management cost was the equivalent to one visit to the psychiatrist plus medication administered for the event (biperidene 2 mg/8 h during three months) [33]

Weight gain management cost was equivalent to one visit to a general physician every three months; this definition was based on expert opinion.

The cost of diabetes [34,35] and its complications (amputation [36], fatal myocardial infarction [37], heart failure [38], ischaemic heart disease [39], non-fatal myocardial infarction [37] and nonfatal stroke [40]) were obtained from literature.

### Unitary costs

All costs were uplifted to 2009 prices, using appropriate price inflation rates [41]. The unitary costs detailed in Table 4 were obtained from the literature and from a Spanish database on health costs [42].

**Table 4:** Unitary cost (€, 2009)

Status	Cost (€)
Relapse (hospital cost)	5,589 (1) [32]
Acute EPS (extra-pyramidal symptoms)	85 (2)
Psychiatric medical visit	68 [42]

Biperidene (2 mg/8 h/day)	17 [31]

Weight gain	117 (3)

General physician medical visit	39 [42]

Diabetes Mellitus (control)	855 [34,35]
Amputation	5,857 [36]
Fatal myocardial infarction	1,531 [37]
Heart failure	4,189 [38]
Ischaemic heart disease	2,474 [39]
Non-fatal myocardial infarction	21,610 [37]
Non-fatal Stroke	5,199 [40]

(1) = including inpatient stay and pharmacological treatment

(2) = including medication (biperidene 2 mg/8 hours/day for three months) plus one medical visit to a psychiatrist

(3) = one visit to a general physician (primary care) for three months

### Sensitivity analysis

One-way deterministic analyses were carried out to test the model's robustness.

One-way deterministic analysis included modifications of the following inputs: relapse probabilities and treatment discontinuation probabilities (for adverse events or for other reasons) that were varied by ± 20%.

## Results

### Deterministic analysis

**Figure 2:**
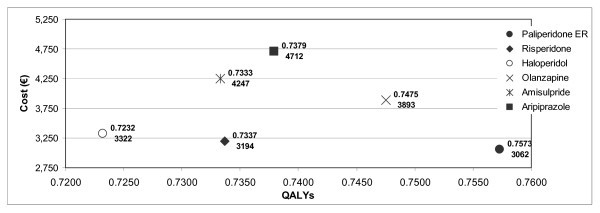
**Cost-effectiveness of antipsychotics for relapse prevention**. Data shown in the figure for each strategy refer to effectiveness in QALYs (upper value) and annual cost (€) (lower value).

Total cost per patient and effectiveness measured in QALYs for the six antipsychotic drugs assessed are shown in Figure 2. Paliperidone ER was the option that yielded more QALYs per patient (0.7573), followed by olanzapine (0.7475), aripiprazole (0.7379), risperidone (0.7337), amisulpride (0.7333) and haloperidol (0.7232). In addition, paliperidone ER was the least costly strategy (€3,062), followed by risperidone (€3,194), haloperidol (€3,322), olanzapine (€3,893), amisulpride (€4,247) and aripiprazole (€4,712), in increasing order.

Average cost-effectiveness ratios of these antipsychotic medications are included in Table 5. Paliperidone ER had the lowest average cost-effectiveness ratio (€4,043 per QALY) and aripiprazole the highest (€6,386 per QALY)

**Table 5:** Average cost-effectiveness (CE) and incremental cost-effectiveness (ICE) ratio of assessed antipsychotics versus haloperidol (€, 2009)

Strategy	Average CE ratio (€/QALY)	ICE ratio (€/QALY gained) versus haloperidol
Amisulpride	5,792	91,584

Aripiprazole	6,386	94,558

Haloperidol	4,593	NA

Olanzapine	5,208	23,621

Paliperidone ER	4,043	Dominant

Risperidone	4,353	Dominant

Dominant: strategy with higher effectiveness (QALYs) and lower cost

#### Incremental Cost-Effectiveness Ratio

**Figure 3:**
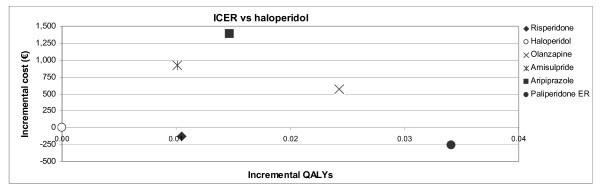
**Incremental cost-effectiveness ratio (ICER) of assessed treatment options versus haloperidol**. QALY: Quality-Adjusted Life Year.

Figure 3 graphically represents the incremental cost-effectiveness of assessed antipsychotics versus the primary comparator (haloperidol). Table 5 show ICERs of assessed antipsychotics versus haloperidol.

Paliperidone ER and risperidone yielded more QALYs and less cost than haloperidol Thus, they were considered dominant strategies, while haloperidol was a dominated strategy.

Considering the common threshold accepted in Spain (€30,000 per QALY gained) [27], olanzapine could also be considered a cost-effective option compared to haloperidol.

Both amisulpride and aripiprazole were above the threshold when compared with haloperidol.

### Sensitivity analysis

Table 6 show details of the deterministic analysis performed. Risperidone is always a dominant strategy versus haloperidol. Paliperidone ER is also a dominant strategy over haloperidol apart from the scenario where basecase probability of relapse is reduced by 20%, which yielded an ICER of €1,687 per QALY gained.

**Table 6:** Deterministic Sensitivity Analysis (ICERs of assessed antipsychotics versus haloperidol)

Parameter	Incremental Cost (€)	Incremental QALYs	ICER versus haloperidol (€/QALY gained)
**Base case (BC)**			

Amisulpride	925	0.0101	91,584

Aripiprazole	1,390	0.0147	94,558

Olanzapine	574	0.0243	23,621

Paliperidone ER	-260	0.0341	Dominant

Risperidone	-128	0.0105	Dominant

**Probability of relapse +20% BC**			

Amisulpride	795	0.0099	80,303

Aripiprazole	1,227	0.0144	85,208

Olanzapine	317	0.0258	12,287

Paliperidone ER	-632	0.0373	Dominant

Risperidone	-279	0.0107	Dominant

**Probability of relapse -20% BC**			

Amisulpride	986	0.0150	65,733

Aripiprazole	1,495	0.0131	114,122

Olanzapine	773	0.0238	32,479

Paliperidone ER	55	0.0326	1,687

Risperidone	-35	0.0099	Dominant

**Probability of discontinuation due to AE +20% BC**			

Amisulpride	861	0.0097	88,763

Aripiprazole	1,356	0.0130	104,308

Olanzapine	516	0.0238	21,681

Paliperidone ER	-287	0.0319	Dominant

Risperidone	-184	0.0099	Dominant

**Probability of discontinuation due to AE -20% BC**			

Amisulpride	930	0.0104	89,423

Aripiprazole	1,373	0.0163	84,233

Olanzapine	575	0.0249	23,092

Paliperidone ER	-291	0.0362	Dominant

Risperidone	-141	0.0114	Dominant

**Probability of discontinuation due to other reasons +20% BC**			

Amisulpride	868	0.0094	92,340

Aripiprazole	1,358	0.0135	100,593

Olanzapine	490	0.0243	20,165

Paliperidone ER	-364	0.0349	Dominant

Risperidone	-211	0.0100	Dominant

**Probability of discontinuation due to other reasons -20% BC**			

Amisulpride	923	0.0105	87,905

Aripiprazole	1,363	0.0157	86,815

Olanzapine	601	0.0242	24,835

Paliperidone ER	-213	0.0332	Dominant

Risperidone	-103	0.0106	Dominant

Dominant: strategy with higher effectiveness (QALYs) and lower cost

## Discussion

To our knowledge, this analysis is the first economic evaluation assessing antipsychotics for prevention and treatment of schizophrenia relapses in Spain.

Atypical antipsychotics are cost-effective when compared with first generation antipsychotics (haloperidol), thus the expected higher acquisition cost of new antipsychotics are generally offset by reductions in other mental healthcare costs, particularly inpatient hospitalisation costs. This indicates that second-generation drugs may be a more efficient choice than traditional antipsychotics [21,43].

Our study showed lower average cost-effective ratios for paliperidone ER, risperidone and haloperidol than for olanzapine, amisulpride or aripiprazole.

In the base case of the present analysis, one year of treatment with paliperidone ER or risperidone compared with haloperidol were dominant strategies. Our results are in line with economic evaluations carried out by other investigators. Risperidone against haloperidol was also a dominant strategy in Canada [44] and had a lower risk of relapse [45]. Treatment with olanzapine resulted in higher costs than risperidone [46], but it was also cost-effective when compared with haloperidol in a Mexican study [47]. In the UK, despite equivalence with respect to the costs of three alternatives, both risperidone and olanzapine were cost-effective when compared with haloperidol due to efficacy gains [48].

Given that the willingness to pay for an additional QALY in Spain is generally estimated to be within the range of €30,000 to €45,000 [25,49] paliperidone ER and risperidone were identified as dominant therapies, olanzapine (€23,621 per QALY) could also be considered to be a cost-effective option versus haloperidol in the schizophrenia relapse treatment.

Modelling based on a decision tree to project 12-month consequences of treatment for different antipsychotic therapies has been previously used in other economics evaluations [50].

As noted in NICE analysis, the key driver of cost effectiveness for antipsychotics is their probability to reduce relapse rates. Indeed, due to lack of head to head trials between the drugs analyzed, the current analysis is built on the mixed treatment comparison built on NICE based on retrospective available data.

The main drivers of the model are probabilities. Data from a systematic review performed by NICE were used, due to the lack of head-to-head trials between the drugs analyzed, although differences in relapse definition over the 17 studies included in this meta-analysis could lead to different relapse rates and potentially influence results.

The one-year treatment period was chosen because most of the clinical trials only assessed efficacy of antipsychotics in a 52-week period, and projection of the efficacy beyond 52 weeks would be a source of bias in the analysis. On the other hand, the last guideline for schizophrenia published by NICE considered a lifetime horizon given the potential need for long-term use of APS drugs by people with schizophrenia in remission.

The primary limitation of this evaluation was the omission of other antipsychotics, such as zotepine and quetiapine. As previously justified, zotepine was not included because it is not marketed in Spain. Quetiapine was also excluded due to the lack of relevant clinical information on this drug related to schizophrenia relapse prevention.

Although antipsychotic medication is associated with a wide range of adverse events, only EPS, weight gain and metabolic effects were considered in our evaluation, as they have been identified to be those with a greater impact on cost-effectiveness ratio.

Following a conservative approach, tardive dyskinesia was not included, despite its long-term and important effects on quality of life, as this event is mainly associated with the primary comparator (haloperidol). Including this side effect could introduce an important bias in the analysis [18].

The quality-adjusted life year (QALY) is routinely used as a summary measure of health outcome for economic evaluation, which incorporates the impact on both the quantity and quality of life [51] QALYs are obtained by multiplying life years gained by an utility value. Utilities represent patient's preferences by a health state, so they are strongly related to cultural aspects. Ideal economic evaluation would be based on data derived from local population where study is performed [52]. However publications with Spanish specific utilities values in the illness of interest are scarce. Although use of values from other areas could be a potential bias' source [53], in the present study, due to the lack of published data related to Spanish population, utilities from UK where used for remission, relapse, acute EPS and weight gain.

According the results from the deterministic sensitivity analysis developed, the model seems to be quite robust, but probabilistic sensitivity analyses would have provided additional information to validate this point.

Despite these limitations, the assumptions considered in the present model appeared to be reasonable and conservative, and the results of the sensitivity analyses indicated the robustness of the findings.

The current analysis shows that relapse prevention is the key driver for cost-effectiveness of antipsychotics in Spain. This conclusion is fully aligned with the results of the NICE analysis in the UK, which acknowledges that drug acquisition costs do not determine the relative cost-effectiveness of antipsychotic medications. Indeed, antipsychotic drugs that reduce the rate and duration of hospital admissions may be cost-saving options in the long-term, despite higher acquisition costs.

With respect to other Spanish studies, our results are also aligned with a previous analysis concerning the costs of schizophrenia in Spain, which showed that drugs account for only 24% of medical costs of the disease in Spain, whereas hospitalization accounted for 73% of those costs [54].

A previous investigation in Spain has shown that number of relapses is directly related to higher treatment costs [20]. The choice of the best alternative to reduce relapses, in efficacy terms, could indeed impact in the total schizophrenia cost. According our results paliperidone ER is the most effective and the least costly strategy.

Our findings may be of interest to clinicians and others interested in outcomes and cost of mental health services among patients with schizophrenia.

## Conclusions

In our analysis, paliperidone ER and risperidone were shown to be dominant antipsychotic therapies compared to haloperidol in Spain. However, as pointed out by NICE, it is worthwhile to highlight that schizophrenia is a highly incapacitating disease and choosing the most appropriate drug and formulation for a particular patient is crucial. The prevention of relapses is a key factor in the efficiency of the antipsychotics.

## Competing interests

This work was supported by an unrestricted research grant sponsored by Janssen-Cilag at Cátedra de Economia de la Salud y Uso Racional del Medicamento in University of Malaga The authors have not transmitted any conflicts of interest, because the concept, design and development of the model have been carried out independently.

IO and MAC are PORIB employees a consultant company specialized in economic evaluation of health technologies.

## Authors' contributions

AJG conceived of the study and performed a general coordination of the project. LP and ACM have made substantial contributions to conception and model design. LP, ACM and AJG have involved in acquisition of data and analysis. AJG, LP and AJG have played key role in interpretation of the results. IO and MAC validated the assumptions taken in model design, reviewed the results, participating in interpretations of data, and were involved in drafting the manuscript. All the authors have participated sufficiently in the work to take public responsibility for appropriate portions of the content. All of them have reviewed the final version of the manuscript and have given a final permission of the version to be published. All authors read and approved the final manuscript.
